# Urban Form and Extreme Heat Events: Are Sprawling Cities More Vulnerable to Climate Change Than Compact Cities?

**DOI:** 10.1289/ehp.0901879

**Published:** 2010-06-23

**Authors:** Brian Stone, Jeremy J. Hess, Howard Frumkin

**Affiliations:** 1 School of City and Regional Planning, Georgia Institute of Technology, Atlanta, Georgia, USA; 2 National Center for Environmental Health, Agency for Toxic Substances and Disease Registry, Centers for Disease Control and Prevention, Atlanta, Georgia, USA; 3 Emory University School of Medicine and; 4 Rollins School of Public Health, Emory University, Atlanta, Georgia, USA

**Keywords:** climate change, extreme heat events, public health, sprawl

## Abstract

**Background:**

Extreme heat events (EHEs) are increasing in frequency in large U.S. cities and are responsible for a greater annual number of climate-related fatalities, on average, than any other form of extreme weather. In addition, low-density, sprawling patterns of urban development have been associated with enhanced surface temperatures in urbanized areas.

**Objectives:**

In this study. we examined the association between urban form at the level of the metropolitan region and the frequency of EHEs over a five-decade period.

**Methods:**

We employed a widely published sprawl index to measure the association between urban form in 2000 and the mean annual rate of change in EHEs between 1956 and 2005.

**Results:**

We found that the rate of increase in the annual number of EHEs between 1956 and 2005 in the most sprawling metropolitan regions was more than double the rate of increase observed in the most compact metropolitan regions.

**Conclusions:**

The design and management of land use in metropolitan regions may offer an important tool for adapting to the heat-related health effects associated with ongoing climate change.

Extreme heat is an important cause of morbidity and mortality. During an average summer about 400 Americans succumb to extreme heat [Centers for Disease Control and Prevention (CDC) 2002a]. Heat-related deaths tend to occur during heat waves. The 1995 Chicago heat wave killed > 500 people over 5 days (Whitman et al. 1997), and the 2003 European heat wave is estimated to have killed > 70,000 people over a few months (Robine et al. 2008). Risk factors for dying during a heat wave include being very old or very young; being homebound, confined to bed, or unable to care for oneself; being socially isolated; lacking air conditioning; and suffering from psychiatric or cardiopulmonary disease (Bouchama et al. 2007; Naughton et al. 2002). Importantly, most heat wave deaths occur in cities, a long-recognized result of the urban heat island effect (Clarke 1972).

This heat effect is a phenomenon through which cities exhibit higher temperatures than the surrounding countryside. This temperature differential, which can exceed 10°C, results from several factors: loss of vegetation with accompanying loss of evapotranspiration; dark surfaces with low albedo (i.e., surface reflectivity), which absorb and then reradiate heat; building configurations that trap heat; and the concentrated generation of heat from generators, vehicles, and other sources (Oke 1982). Thus, urban form can intensify extreme heat events (EHEs) in cities.

Cities have significantly decentralized over recent decades in a pattern known as urban sprawl. Sprawl features geographic expansion over large areas, low-density land use, low land-use mix, low connectivity, and heavy reliance on automobiles relative to other modes of travel (Squires 2002). This trend has several impacts on health, including reduced physical activity, worsened air pollution, increased risk of motor vehicle injuries, and others (Frumkin et al. 2004). Low-density patterns of land use have also been associated with enhanced surface temperatures in cities (Stone and Norman 2006), raising the prospect that sprawl could have an effect on the probability and intensity of heat waves.

This finding is salient because EHEs in cities have become more common in recent decades (Gaffen and Ross 1998; Gershunov et al. 2009)—a trend that is expected to continue with climate change (International Panel on Climate Change 2007). If urban sprawl contributes to heat waves, it could have implications for heat wave preparedness and could inform decisions about future patterns of urban development.

In this article, we report on the association between urban form and EHEs. Specifically, we tested the hypothesis that sprawling patterns of metropolitan land use are more closely associated with the rate of increase in EHEs over a five-decade period than are compact patterns of metropolitan land use.

## Methods

We examined the correlation between a sprawl index (based on land-use data from 2000) (Ewing et al. 2003a) and the rate of increase in EHEs over a five-decade period (1956–2005). Each of these variables is defined below, followed by a description of the analytical approach.

### Sprawl index for U.S. metropolitan regions

To quantify metropolitan land-use patterns, we made use of a sprawl index developed by Ewing et al. (2003a). Based on four spatial elements of physical form combined through principal components analysis, including the centeredness, connectivity, density, and mix of land uses within metropolitan regions, the sprawl index quantifies the spatial configuration and centralized intensity of land use within 83 of the largest U.S. metropolitan regions based on data from the 2000 Census and other national surveys. Previous work found the index to be a reliable predictor of travel behavior, physical activity, vehicular safety, and air quality across the metropolitan regions for which values are available (Ewing et al. 2003a, 2003b; Stone 2008; Trowbridge and McDonald 2008). We included in our study 53 of the 83 regions for which the sprawl index is available and that are included in the National Climatic Data Center (NCDC) EHEs database [National Oceanic and Atmospheric Administration (NOAA) 2009]. Table 1 describes each component of the composite sprawl index.

### Extreme heat event data

The extreme heat event data used in this study were drawn from a heat stress index maintained for 187 U.S. cities by the NCDC (NOAA 2009). The index is based on a measure of apparent temperature (*A*) that reflects both temperature and humidity and is derived through the following equation:


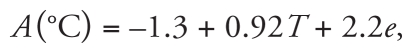


where *T* is ambient air temperature (°C) and *e* is water vapor pressure (kPa) (Steadman 1984).

The NCDC heat stress index, which extends the work of Gaffen and Ross (1998), has classified an extreme heat event as any day in which the minimum, maximum, or average apparent temperature exceeds the 85th percentile of the base period (1961–1990) for each first-order weather station included in the database. Prior work has demonstrated that the 85th percentile of apparent temperature is associated with elevated levels of heat-related mortality (Kalkstein and Davis 1989).

As increasing trends in minimum temperatures are most closely associated with adverse health outcomes (Kalkstein and Davis 1989), we quantified the average annual change in minimum temperature heat events over the most recent 50-year period for which data is available—1956–2005. To be included in the data set, a metropolitan region must have complete data on EHEs for 42 of the 50 years in the study period. For each metropolitan region in the data set, we measured the interannual change in the number of EHEs and then averaged these interannual changes over the full study period to derive the mean annual change in EHEs per region.

### Analysis

To test the hypothesis that urban form is associated with the rate of change in EHEs, we measured the correlation between the mean annual change in the number of EHEs between 1956 and 2005 and the sprawl ranking of each region in 2000. We then performed a *t*-test to gauge the statistical significance of a linear association. Because our interest is in measuring the influence of the spatial pattern of urban development on EHEs rather than population characteristics, we controlled for the influence of metropolitan population size in 2000 and the rate of metropolitan population growth since 1950 on trends in EHEs through the derivation of partial correlation coefficients. It should be noted that geographic variation in regional climates is internally controlled in the extreme heat event measure, which employs region-specific temperature thresholds (i.e., the 85th percentile of a long-term temperature trend for each metropolitan statistical area) to identify extreme temperature episodes.

We used SPSS (version 16; SPSS, Chicago, IL) to perform all statistical analyses.

## Results

An analysis of trends in excessively hot days over the period of 1956–2005 found the frequency of EHEs to be increasing significantly on an annual basis. Extending the trend measured by Gaffen and Ross (1998) by an additional decade, our analysis found that the mean annual number of EHEs in major U.S. cities increased by 0.20 days/year [95% confidence interval (CI), 0.14–0.26], which is consistent with 10 more events per city, on average, in 2005 than in 1956.

**Figure 1:**
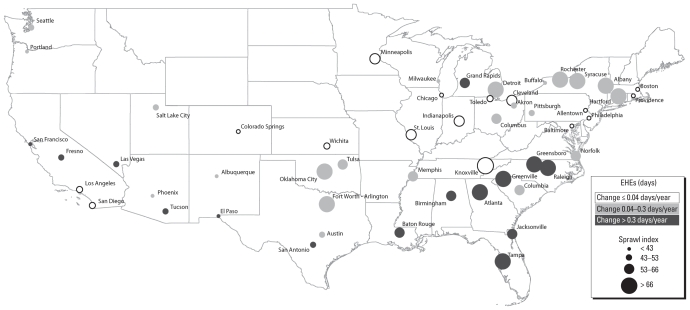
Sprawl ranking and mean annual change in frequency of EHEs by metropolitan statistical area.

**Figure 2:**
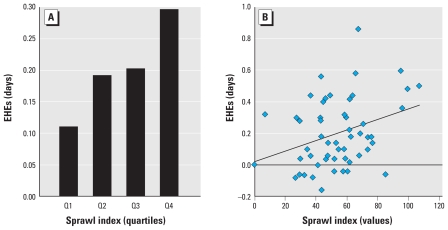
Mean annual change in frequency of EHEs, by sprawl index.

As illustrated in Figures 1 and 2, the rate of increase in annual EHEs over this 50-year period varied significantly by metropolitan form. Although the average annual number of EHEs increased during this period across all cities, the most sprawling cities (top quartile) experienced a rate of increase in EHEs that was more than double that of the most compact cities (bottom quartile). Between 1956 and 2005, the most compact cities experienced an average increase in the number of EHEs of 5.6 days (95% CI, 0.9–10.3), whereas the average annual number of events increased by 14.8 days (95% CI, 7.9–21.7) in the most sprawling cities. Variation in the size or rate of growth in metropolitan populations did not diminish the measured statistical association between land-use patterns and the rate of increase in EHEs in these cities (*r* = 0.34; *p* < 0.05). These findings are consistent with the hypothesis that urban sprawl contributes to EHE frequency.

## Discussion and Conclusions

This analysis yields two principal findings. First, the annual occurrence of EHEs continues to increase in large metropolitan regions of the United States. This finding, previously demonstrated by Gaffen and Ross (1998) for the period 1949–1995, is extended here to 2005 for 53 metropolitan regions for which both apparent temperature and sprawl index values are available. Second, the rate of increase in EHEs is higher in sprawling than in more compact metropolitan regions, an association that is independent of climate zone, metropolitan population size, or the rate of metropolitan population growth.

The mechanisms of EHEs in cities are complex. Cities are typically characterized by lower rates of evapotranspiration and lower albedo than are rural areas, as a result of the reduced vegetative cover and the increased presence of darkly hued bituminous roofing and paving materials. Urban areas are further characterized by higher thermal loads than are rural areas, because of the concentrated presence of generators, air-conditioning units, motor vehicles, and other heat sources. Although our data do not permit an assessment of the relative contribution of each of these factors, the loss of vegetative cover is well established as a principal driver of the urban heat island effect (Oke 1982; Stone and Norman 2006). The availability of data on rates of deforestation across the continental United States between 1992 and 2001 enables an assessment of the association between changes in regional vegetative cover over time and EHEs during a portion of our study period.

The U.S. National Land Cover Database provides maps of 21 categories of land cover across the continental United States between 1992 and 2001 (U.S. Department of the Interior 2009). For each of the 53 metropolitan regions in our study, we measured the area of forest canopy change over this 10-year period and associated it with the sprawl index and rate of change in EHEs. The results of this analysis indicate that the rate of deforestation in the most sprawling metropolitan regions is more than double the rate in the most compact metropolitan regions. For those regions in the top quartile of the sprawl index, 187 km^2^ (95% CI, 33.4–339.8) of forest were lost during this decade, compared with 72 km^2^ (95% CI, 1.4–143.3) for those regions in the bottom quartile of the sprawl index. This analysis further finds the rate of tree canopy loss to be significantly associated with the rate of increase in EHEs over time, when controlling for metropolitan population size and growth rate (*r* = 0.30; *p* < 0.05). Based on this assessment, there is evidence to suggest that sprawling patterns of urban development may be influencing the frequency of EHEs through their effects on regional vegetative land cover.

The mechanisms through which extreme heat translates into human health effects are also complex. The incidence of heat-related illness in the United States has been level or slightly declining despite rising average temperatures since roughly 1980, with significant variability depending on incidence of heat waves [Centers for Disease Control and Prevention (CDC) 1995, 1999, 2002b]. This relatively stable mortality rate is presumably due to increased prevalence of protective factors such as air conditioning. Differences in the incidence of heat-related morbidity and mortality between sprawling and compact cities have not been examined, and our data do not allow for such a comparison. It is possible that protective factors have thus far outweighed the influence of urban form on the incidence of heat-related illness. Projecting forward, however, the exposure amplification associated with sprawl may be increasingly important as average ambient temperatures continue to climb and eventually outpace physiologic adaptation thresholds in many regions. Indeed, as Shanghai’s urban heat island has grown, heat-related mortality rates have increased. This finding suggests that there is the potential for a similar trend in association with urban sprawl (Tan et al. 2010)—a question that deserves further study.

Our findings have clear implications for public health officials and urban planners. Most important, there is a need to incorporate land-use patterns into models that project climate change impacts over time in urban areas. Anticipating the increased exposure to extreme heat in cities, planners can work to control extreme temperatures through such strategies as preservation of regional green space; the installation of street trees, more reflective surfaces on roads and buildings, and green roofs; and replacement of vehicular travel by transit, walking, and bicycling—features all promoted through more compact design. Models suggest that urban albedo and vegetation enhancement strategies have significant potential to reduce heat-related health impacts (Silva et al. 2010). These risk reduction strategies must be complemented by strategies that identify and protect vulnerable populations, standard elements of heat-wave preparedness plans (Bernard and McGeehin 2004).

Such strategies, fortunately, do more than reduce the risk of heat waves. Sprawl is associated with a wide range of adverse exposures, including ozone exceedances (Stone 2008), poor water quality (Tu 2007), and adverse health outcomes from obesity (Ewing et al. 2003b; Lopez 2004) to decreased physical activity (Garden and Jalaludin 2009; Rashad 2009) to fatal road traffic injuries (Ewing et al. 2003c; Lucy 2003; Schlundt et al. 2004). Interventions that increase density, green space, and public transit offer considerable co-benefits by reducing air pollution levels and the risk of injuries and promoting physical activity (Younger et al. 2008). They also increase urban resiliency to other climate-related risks such as severe precipitation events; trees, for example, play a key role in managing stormwater runoff and flooding. With the increasing frequency and severity of environmental hazards such as heat, urban design strategies will play an important role in reducing vulnerability, promoting health, and building resilience.

## Figures and Tables

**Table 1:** Derivation of the sprawl index.

Attribute	Derivation
Centeredness	The centeredness variable is a measure of the degree of mono- or polycentrism within a metropolitan region and is based on three indicators: a density gradient, the percentage of the metropolitan population within a fixed radius of the central business district, and the number of population centers as defined by proximity of census tracts to regional density maxima.
Connectivity	The connectivity variable is a measure of the density of the street network and was based on the average block size and the percentage of blocks less than approximately 500 feet on a side (consistent with the dimension of a traditional urban block). As block size increases, the number of street intersections per unit of area decreases, which serves as an indicator of street network density.
Density	A composite density factor was derived through principal components analysis incorporating measures of gross population density, the proportion of metropolitan populations living at very low or very high densities, and the proximity of census tracts to urban centers.
Land-use mix	Three elements of land-use mix were integrated into a single, composite measure through principal components analysis. These elements include the ratio of jobs to population, the diversity of land uses, and the accessibility of residential uses to nonresidential uses at the level of the transportation analysis zone and within a 1-mile radius.
Sprawl index	A composite measure of urban compactness or sprawl was developed through an integration of these four urban form factors through principal components analysis.

Each urban form attribute is reported on a scale with a mean value of 100 and an SD of 25 [across the 83 regions included in the Ewing et al. study (2003a)]. Higher values of the scores for centeredness, connectivity, density, and land-use mix reflect higher intensities of these attributes. Note that the Ewing et al. sprawl index scales in a negative direction (i.e., higher scores denote lower levels of sprawl) and has been modified in this study to scale in a positive direction (i.e., higher scores denote higher levels of sprawl) for ease of interpretation. The table is adapted from Stone (2008) and based on description of sprawl index from Ewing et al. (2003a).
